# Bio-Based Grease from Agricultural Waste: Modified Cellulose from Corn Stover for Sustainable Lubrication

**DOI:** 10.3390/ma18184413

**Published:** 2025-09-22

**Authors:** Yuhao Fang, Gaobo Lou, Qiang Wu, Xingguo Cheng, Yifan Chen

**Affiliations:** 1 College of Chemistry and Materials Engineering, Zhejiang A&F University, Hangzhou 311300, China; 18839969561@163.com; 2 National Engineering and Technology Research Center of Wood-Based Resources Comprehensive Utilization, Zhejiang A&F University, Hangzhou 311300, China; 3 Hangzhou Derunbao Grease Co., Ltd., Hangzhou 311305, China; chengxingguo@derunbao.com

**Keywords:** lubrication grease, bio-based, rheology, tribology

## Abstract

In this study, a green lubricating grease was prepared based on cellulose and epoxidized soybean oil (ESO). The cellulose extracted from the corn stover was functionalized using diphenylmethane diisocyanate (MDI), which enhances its compatibility and thickening ability in non-polar oil, and subsequently dispersed in ESO to form a stable gel-like bio-based grease. The functionalized surface of cellulose was characterized by FTIR, SEM, and XRD. And the rheological and tribological characteristics of the prepared bio-based grease were discussed. The superior lubricity and anti-wear properties of our bio-based grease are demonstrated by its lower friction and diminished wear relative to commercial lithium-based formulations. This work provides practical guidance for designing environmentally friendly grease for sustainable lubrication.

## 1. Introduction

With the development of industrialization, traditional greases based primarily on mineral oils and metal soap thickeners have been widely employed in machinery applications [1,2,3,4]. Nevertheless, these greases not only cause environmental pollution during production and use, but they also pose significant challenges to the sustainability of resources [5,6]. Therefore, in response to growing global concerns regarding climate change and environmental protection, it is increasingly recognized that traditional greases are insufficient to meet the demands of green and sustainable development [7,8].

Based on this background, bio-based lubricating grease has attracted extensive attention owing to its environmentally friendly nature and renewability [9,10]. Bio-based lubricating greases, which are derived from biomass, have been demonstrated to alleviate dependence on petroleum resources and present notable advantages, such as enhanced biodegradability and diminished environmental impact. Consequently, these greases contribute to a reduction in greenhouse gas emissions and facilitate the transition toward a circular economy [11,12,13]. Thus, various works on bio-based lubricating greases have been reported in recent years. Gallego [14] modified the surface of methyl cellulose using hexamethylene-diisocyanate (HMDI) and prepared an all-bio-based grease utilizing castor oil. Their findings demonstrated that one end of the -NCO group of the HMDI molecule reacted with cellulose -OH, while the other end reacted with -OH in castor oil, enhancing the cross-linking within the grease structure. Sanchez [15] formulated several bio-based greases using soybean oil and castor oil as base oils and chitin, chitosan, and their acylated derivatives as thickening agents. In tribological tests, the friction coefficients ranged from 0.25 for chitin-based grease to 0.12 for acylated chitin-based lubrication. Rheological tests revealed that the SAOS function values of all greases increased with thickener concentration. In addition. Wu [16] extracted lignin from beech using organic solvents, modified it with a silane coupling agent, and synthesized lignin-based grease employing castor oil. The friction coefficient of the grease initially decreases, followed by an increase with the concentration of modified lignin. High-temperature oxidation differential scanning calorimetry (DSC) tests indicated that while the oxidation induction time of castor oil was 20 s, that of the modified lignin grease was 1959 s. These studies demonstrate the enormous potential of using bio-based raw materials to prepare sustainable lubricants.

Corn straw, as an agricultural waste, has not been well utilized. Burning straw annually not only causes air pollution but also poses a fire hazard. The extraction and utilization of cellulose from corn stover offer a promising strategy to mitigate the environmental burden of agricultural waste while supplying a high-value renewable biomass resource [17,18]. The compatibility of cellulose with base oil and its thickening capacity can be significantly enhanced through isocyanate modification [14,19,20,21]. Epoxidized soybean oil (ESO) is selected as the base oil of bio-based lubricating grease due to its notable oxidative stability and biodegradability [22,23]. In summary, this work developed an all bio-based lubricating grease using renewable resources, with isocyanate-modified cellulose as the thickener and ESO as the base oil. This research aims to provide an innovative strategy and technological pathway for the sustainable development of the lubricating grease industry and to contribute to the alleviation of environmental pressures.

## 2. Materials and Methods

### 2.1. Materials

The following materials were used: corn straw (St) purchased in Jiangsu, China; epoxidized soybean oil (ESO, CP) and triethylamine (C_6_H_15_N, AR) purchased from Aladdin Reagent Co., Ltd. (Shanghai, China); hydrogen peroxide (H_2_O_2_, 30%), purchased from XiLong Scientific (Shenzhen, China); hydrogen chloride (HCl 36.46%) purchased from Yonghua Chemical Co., Ltd. (Changshu, China); toluene (C_7_H_8_, 99.5%) and anhydrous ethanol (C_2_H_6_O) purchased from Sinopharm Chemical Reagent Co., Ltd. (Shanghai, China); and diphenylmethane diisocyanate (MDI, 98%) purchased from Shanghai Maclin Biochemical Technology Co., Ltd. (Shanghai, China).

### 2.2. Cellulose Extraction

Corn stover was rinsed with deionized water, pressed to remove the water, and then dried in an oven at 120 °C for 24 h. The dried corn stover was then ground and sieved to a 30-mesh size to obtain the straw powder (St). A total of 6 g of stover powder was added to a mixture of 40 mL of toluene and 20 mL of C_2_H_6_O, and the mixture was stirred at 100 °C for 6 h. After the mixture reached room temperature, it was filtered and washed with C_2_H_6_O, followed by drying at 60 °C. The straw powder was then added to 100 mL of 0.1 mol/L HCl solution and stirred at 85 °C for 2 h. Once cooled, the mixture was filtered and washed with deionized water until neutral, followed by drying at 60 °C. To obtain pure cellulose (St-C), the straw powder was added to a mixture of 100 mL of 6% NaOH solution and 0.7% H_2_O_2_ solution and stirred at 80 °C for 2 h. After cooling, the mixture was filtered and washed with deionized water until neutral and then dried using a freeze dryer.

### 2.3. Cellulose Modification

Quantities of 9 g of cellulose, 6.25 g of MDI, and 0.5 g of triethylamine were added to 150 mL of toluene solution, with the triethylamine acting as the catalyst. The reaction proceeded under continuous stirring in a nitrogen atmosphere at ambient temperature for 24 h. Subsequent to the completion of the reaction, the mixture was filtered and rinsed with toluene 3~5 times to remove unreacted MDI and triethylamine. Finally, the isocyanate-modified cellulose (St-C-MDI) was obtained by drying at 60 °C for 24 h.

### 2.4. Grease Preparation

In a beaker, 40 g ESO was heated to 80 °C, 10 g St-C-MDI was added, and the temperature was raised to 150 °C. The mixture was stirred for 30 min to ensure complete expansion. Having been cooled to room temperature, the product was milled 2 to 3 times to obtain a homogeneous grease (St-C-MDI/ESO).

### 2.5. Characterizations

To analyze the surface microstructure of modified cellulose, a scanning electron microscope (SEM, Hitachi SU 8010, Tokyo, Japan) was employed. Elemental composition was assessed using energy-dispersive X-ray spectroscopy (EDS), while functional groups and chemical structures were characterized via Fourier-transform infrared spectroscopy (FTIR, Brucker Vertex-70, Bremen, Germany). The crystalline structure of cellulose samples was investigated using an X-ray diffractometer (XRD, Smart Lab 9, Tokyo, Japan).

### 2.6. Thermal Stability

The thermal stability of the samples was evaluated using a TA Q500 thermogravimetric analyzer (New Castle, DE, USA) under N_2_ atmosphere by heating from 30 to 800 °C with a ramp rate of 10 °C/min.

### 2.7. Rheological Investigations

The rheological properties of the greases were characterized using an Anton Paar MCR 302 (Graz, Austria) rotary rheometer fitted with a 25 mm diameter parallel plate sensor, with a gap setting of 1 mm. Small-Amplitude Oscillatory Shear (SAOS) testing was conducted within the linear viscoelastic region, with shear rates spanning from 10^−1^ to 10^2^ rad/s. The viscoelastic behavior of the greases was quantified to ascertain the flow point within the strain range of 1% to 100%. The influence of strain conditions on the viscoelastic properties was elucidated through a recovery test that comprised three distinct phases: initially, the oscillatory strain amplitude of 0.1% was held for 600 s; subsequently, the sample was subjected to a strain of 5% for an equivalent period; and ultimately, the conditions were replicated to match those of the initial phase.

### 2.8. Tribological Tests

The tribological performance of the grease was evaluated using a four-ball friction testing machine (MS-10JS, Shenzhen, China). Tests were conducted following ASTM-D4172 [24] under a load of 40 kgf (392 N), at 1200 r/min, and for 60 min. The wear scar diameter on the steel balls was measured using an optical microscope.

### 2.9. Antioxidation Test

The antioxidation test was evaluated according to SH/T 0790-2007 [25] using the NETZSCH FPB-O DSC instrument (Selb, Germany) under pure oxygen atmosphere at 3.5 MPa.

## 3. Results and Discussion

### 3.1. Isocyanate Modification of Cellulose

**Figure 1:**
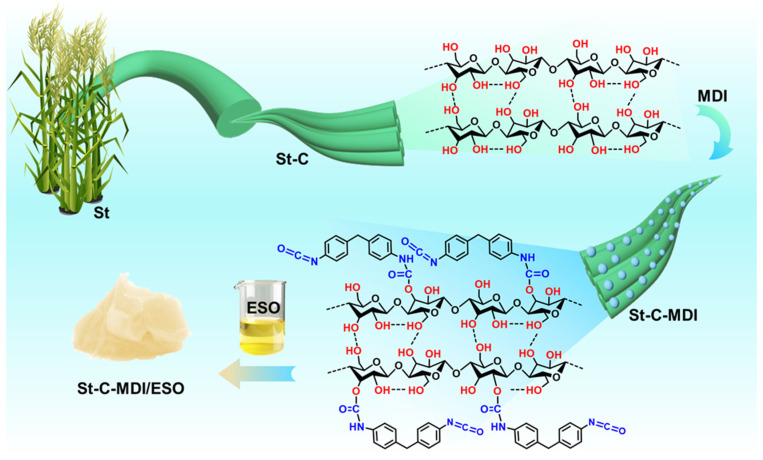
Grease preparation process.

Figure 1 outlines the procedure for preparing the grease. Through a chemical treatment, hemicellulose and lignin are removed from corn stalks to obtain pure cellulose. As a complex polysaccharide, cellulose consists of glucose units connected via β-1,4-glycosidic bonds, and its molecular structure contains a multitude of hydroxyl groups (-OH) [26,27,28]. These hydroxyl groups reduce the compatibility of cellulose with the base oil. The surface of cellulose is modified with isocyanate groups (-NCO) [20,29] which involves the reaction between cellulose hydroxyl groups and isocyanate groups to form urethane linkages, thereby reducing the hydroxyl content and increasing the compatibility with the base oil.

**Figure 2:**
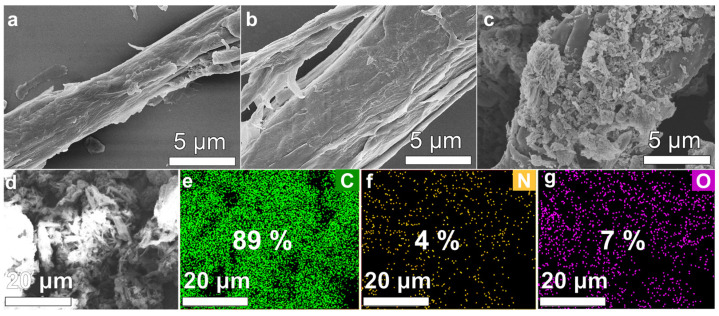
SEM photographs of (**a**) St, (**b**) ST-C, and (**c**) St-C-MDI; (**d**–**g**) element mapping images of St-C-MDI.

Figure 2a–c present the SEM images of St, St-C, and St-C-MDI. The surface of St appears relatively smooth, while the surface of St-C exhibits numerous isolated fibrils. The morphological change results from the removal of lignin and hemicellulose, which separates the cellulose from the overall structure. The surface of St-C-MDI displays a three-dimensional porous structure, which enhances the adsorption capacity and contributes to the thickening effect. The EDS results of St-C-MDI are shown in Figure 2d–g, with the uniform distribution of C, O, and N elements on the surface of St-C-MDI confirming the successful grafting of MDI onto the cellulose surface.

**Figure 3:**
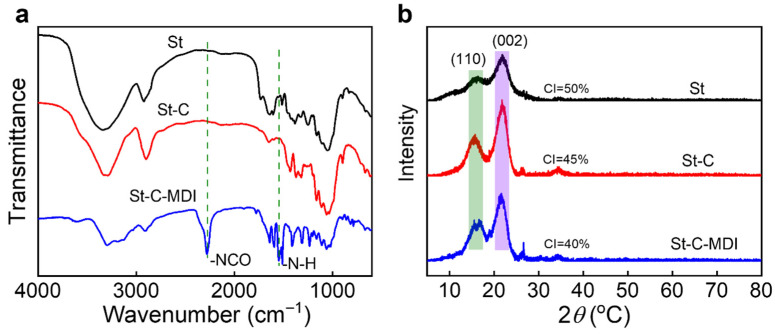
(**a**) FTIR and (**b**) XRD spectra of St, St-C, and St-C-MDI.

Figure 3a displays the FTIR spectra of St, St-C, and St-C-MDI. New absorption bands emerge in the St-C-MDI spectrum at 2280 cm^−1^ and 1540 cm^−1^, which are attributed to residual -NCO groups from MDI and newly formed -N-H bonds, respectively, resulting from the reaction between -NCO and cellulose hydroxyl groups [30,31]. The XRD patterns exhibit characteristic diffraction peaks at 2θ = 16.5° and 22.5°, corresponding to the cellulose (110) and (200) crystal planes, respectively [32,33]. The crystallinity index (CI) was subsequently determined using Equation (1):

(1) $$CI\left(\%\right)=\frac{I_{c}}{I_{c}+{KI}_{a}}\times 100$$

where *I_c_* is the integral intensity of the diffraction from the crystalline regions, *I_a_* represents the integral intensity of the diffraction from the amorphous regions, and *k* is the relative scattering factor between the crystalline and amorphous parts per unit substance. The crystallinity indices for St, St-C, and St-C-MDI were found to be 50%, 45%, and 40%. The extraction process of cellulose, which involves the elimination of lignin and hemicellulose that resulted in the destruction of the crystal regions, thus results in a lower crystallinity for St-C compared to St. Additionally, the isocyanate modification is achieved via a nucleophilic addition reaction, in which hydroxyl groups from the cellulose surface react with isocyanate groups of MDI to form urethane linkages, and leads to a decrease in cellulose crystallinity. The FTIR and XRD characterization spectra confirm the successful grafting of MDI onto the surface of cellulose.

**Figure 4:**
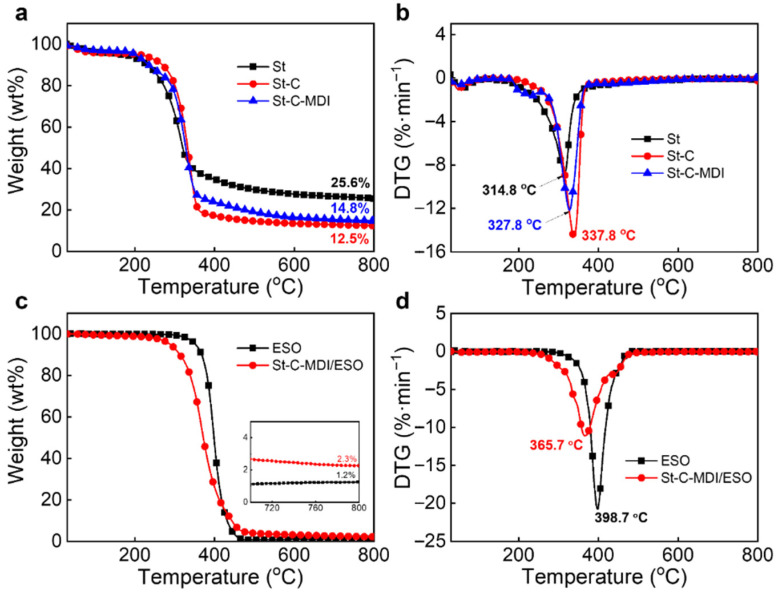
TG and DTG spectra of (**a**,**b**) St, St-C, and St-C-MDI and (**c**,**d**) ESO and St-C-MDI/ESO.

Figure 4 presents the thermal decomposition profiles of the cellulose and greases under a nitrogen atmosphere, and the relevant data are summarized in Table 1. As displayed in Figure 4a,b, the char yield (*Y_c_*) of St-C is significantly lower compared to St, which can be attributed to lignin that possesses a high carbonization ability being removed from St-C.

In addition, the *Y_c_* of the modified St-C-MDI increases compared to that of St-C, which is ascribed to the formation of a dense char layer resulting from the MDI grafted onto the cellulose surface. This layer slows down further thermal decomposition of cellulose, leading to a higher *Y_c_* for St-C-MDI. The maximum thermal decomposition temperature (*T_max_*) of the modified St-C-MDI is 327.8 °C, slightly lower than that of St-C at 337.8 °C. This reduction in thermal stability is mainly due to the urethane bonds formed between MDI and cellulose, which are prone to cleavage at high temperatures. Figure 4c,d compare the thermal decomposition behaviors of St-C-MDI/ESO and ESO. The degradation behavior of St-C-MDI/ESO is similar to that of ESO, which is expected since the base oil constitutes 80% of the grease and is a dominant behavior. However, the *Y_c_* of St-C-MDI/ESO (2.3%) is higher than that of ESO (1.2%), indicating that the St-C-MDI forms a cross-linked network within the base oil, thereby enhancing the overall thermal stability of the grease [34].

**Figure 5:**
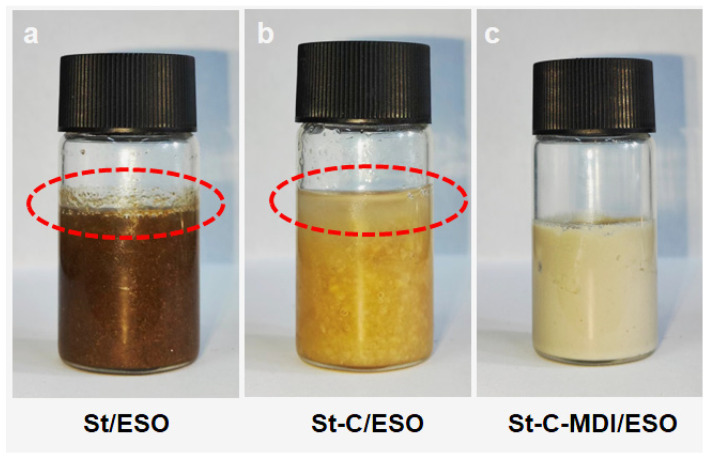
The photos of (**a**) St-C-MDI/ESO, (**b**) St/ESO, and (**c**) St-C/ESO after being left to stand for 14 days; Red circle: Separation occurs.

Two other comparison greases, St/ESO and St-C/ESO, were prepared by incorporating equal proportions of St and St-C into ESO. After 14 days of static storage, as shown in Figure 5, both St/ESO and St-C/ESO displayed severe phase separation, whereas St-C-MDI/ESO retained its homogeneous structure. This result indicates that both raw straw powder and unmodified extracted cellulose lack sufficient thickening capacity for use as grease thickeners. Consequently, a commercial lithium-based grease (Li/MO) without additives was employed as a reference sample for further comparison.

### 3.2. Rheological Characterization

Rheological properties describe the response of grease to applied stress or strain, reflecting changes in its flow behavior and structural deformation. Specifically, these properties characterize how the viscosity of grease varies with shear rate and oscillation frequency [35,36,37,38]. These properties are essential for guiding the development and application of greases. Therefore, a series of rheological tests was conducted on St-C-MDI/ESO and Li/MO using an Anton Paar MCR 302 rotary rheometer (Graz, Austria).

#### 3.2.1. Small-Amplitude Oscillatory Shear (SAOS)

**Figure 6:**
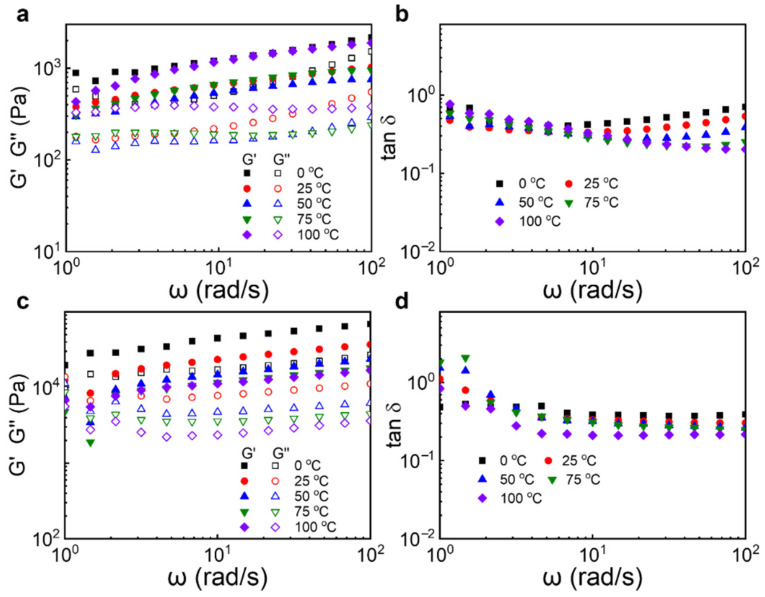
SAOS results of (**a**,**b**) St-C-MDI/ESO and (**c**,**d**) Li/MO at different test temperatures.

The viscoelastic properties of the greases within the linear viscoelastic region under various temperatures were investigated using SAOS tests [39,40,41], with the results depicted in Figure 6. Figure 6a presents the SAOS response curves for St-C-MDI/ESO, where the loss modulus (G″) is significantly lower than the storage modulus (G′) at all temperatures. This indicates that the elastic behavior dominates over the viscous behavior when St-C-MDI/ESO is subjected to shear, implying that it can effectively store energy and return to its original shape after the removal of external forces without significant energy loss. The tangent of the phase angle (tan δ) is given by G″/G′, where G″ and G′ represent the loss modulus and storage modulus, respectively. This reflects the material’s viscoelastic behavior by revealing the phase difference between stress and strain under cyclic loading [42,43]. As shown in Figure 6b, both temperature and frequency affect the tan δ values of St-C-MDI/ESO. Below 75 °C, the tan δ values decrease initially and then increase with increasing frequency, indicating a transition from elastic to viscous behavior as the frequency rises. This is attributed to the sensitivity of the grease’s internal molecular relaxation processes to frequency. At low frequencies, the thickener molecules or structural units within the grease have sufficient time to respond to the external force and rearrange, leading to energy dissipation and higher viscous characteristics, resulting in larger tan δ values. As the frequency increases, the time for molecular rearrangement decreases, reducing the energy dissipation and thus decreasing tan δ values. At high frequencies, the molecules cannot respond to the rapidly changing forces, resulting in a reduction in the storage modulus (G′) and a rise in the loss tangent (tan δ). Above 75 °C, the tan δ values of St-C-MDI/ESO show a continuous downward trend with increasing frequency, suggesting that elastic behavior remains dominant.

The SAOS response curves for Li/MO are shown in Figure 6c; similar to St-C-MDI/ESO, Li/MO exhibits a G′ value significantly higher than G″ across all temperatures, which is characteristic of the rheological behavior of grease. The tan δ values of Li/MO decrease with increasing temperature, which is attributed to enhanced molecular motion and elevated intermolecular friction. This results in an increase in G″ and a corresponding decrease in tan δ values.

#### 3.2.2. Viscosity Test

**Figure 7:**
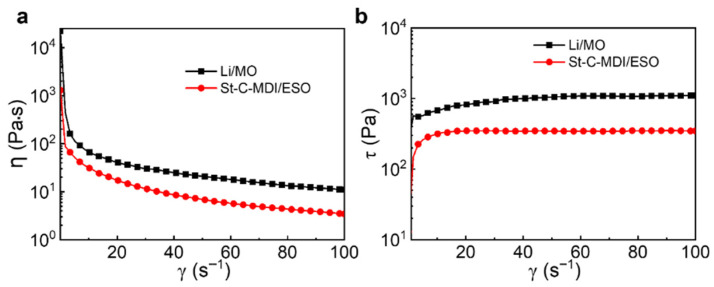
The variation in (**a**) shear viscosity and (**b**) shear stress with shear rates for St-C-MDI/ESO and Li/MO.

Figure 7a,b present the variation curves of shear viscosity (η) and shear stress (τ) for the greases as a function of shear rate (γ). As observed in Figure 7a, the shear viscosities of both St-C-MDI/ESO and Li/MO exhibit a distinct shear-thinning behavior, marked by a gradual decline in shear viscosity with increasing shear rate, which is consistent with gel-like rheological properties [44,45].

Figure 7b shows that the shear stress of both St-C-MDI/ESO and Li/MO increases with shear rate. The trend is more pronounced at low shear rates and tends to stabilize at high shear rates. Such behavior can be attributed to the modified cellulose within the grease, which requires a finite time to respond to adapt and reorganize under shear stress. At low shear rates, the thickener molecules have more time to realign, leading to a more significant increase in shear stress. As the shear rate rises, the available response time for the modified cellulose shortens, leading to a gradual stabilization of the shear stress. A similar trend is observed for Li/MO.

#### 3.2.3. Viscoelasticity Test

**Figure 8:**
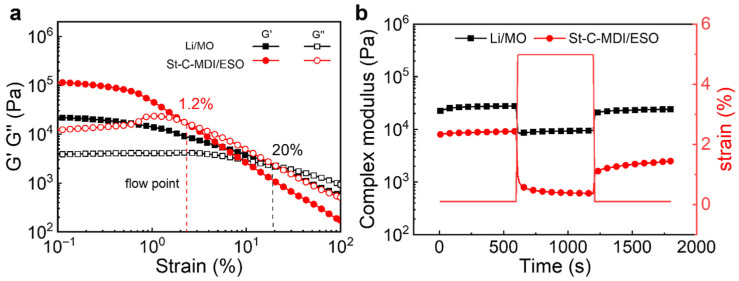
(**a**) Strain sweep and (**b**) structural recovery for St-C-MDI/ESO and Li/MO.

Under viscoelastic testing conditions, the G′ and G″ of grease are two critical parameters whose variation patterns can reflect the rheological behavior and structural transitions of the grease [46,47]. As depicted in Figure 8a, within the linear viscoelastic region, both G′ and G″ maintain stable values that do not depend on strain amplitude, indicating a proportional relationship between stress and strain in the grease. Beyond a critical strain value, the structure of the grease is disrupted, leading to a decrease in G′ and an increase in G″ and eventually converging at the flow point. This intersection point indicates the transition from elastic to flow behavior in the grease. G″ surpasses G′ when the strain goes beyond the flow point, which signifies that the energy dissipated in viscous flow exceeds the energy stored in elastic deformation, resulting in grease flow. It is observed that the flow point of St-C-MDI/ESO (1.2%) is lower than that of Li/MO (20%), suggesting a lower structural strength in St-C-MDI/ESO.

#### 3.2.4. Structural Recovery

The structure will be destroyed when grease is subjected to deformation beyond the linear viscoelastic region, and by reducing the deformation back to within the linear region, the structure partially recovers [48,49]. Figure 8b illustrates the structural recovery characteristics of the greases, where the complex modulus (G*) is calculated using Equation (2). The percentages of structural destruction and recovery are calculated using Equations (3) and (4), respectively:

(2) $$G^{*}\;=\;\sqrt{{G^{\prime }}^{2}+{G^{\prime \prime }}^{2}}$$

(3) $$\mathrm{Destruction}=\;\frac{G_{0}^{*}-G_{1}^{*}}{G_{0}^{*}}\;100$$

(4) $$\mathrm{Recovery}=\frac{G_{2}^{*}-G_{1}^{*}}{G_{0}^{*}-G_{1}^{*}}\;100$$

where $G_{0}^{*}$ denotes the modulus corresponding to the initial shear strain applied within the linear viscoelastic region; $G_{1}^{*}$ reflects the modulus under a second strain beyond the linear region; and $G_{2}^{*}$ denotes the recovered modulus when initial strain is reapplied within the linear viscoelastic region. The relevant data are presented in Table 2. It is observed that St-C-MDI/ESO exhibits a higher structural destruction and a lower recovery compared with Li/MO, which is primarily due to the lithium soap fibers being more compact and not easy to destroy, and the interaction between fibers makes the recovery performance faster after the structure is destroyed.

### 3.3. Tribological Test

**Figure 9:**
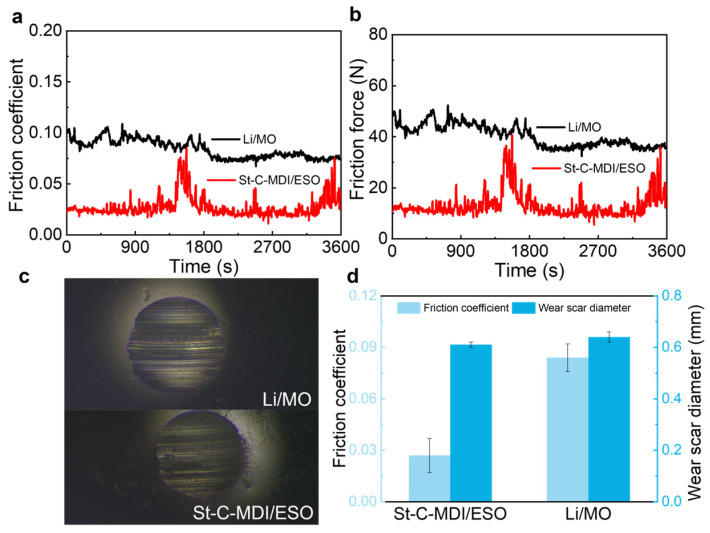
(**a**) Friction coefficient, (**b**) friction forces, and (**c**) wear scar photos of St-C-MDI/ESO and Li/MO, as well as (**d**) a comparison of antifriction and wear-resistant properties.

To evaluate the tribological performance of St-C-MDI/ESO and Li/MO, a four-ball friction tester was employed. The corresponding results are presented in Figure 9, with key data summarized in Table 3. Figure 9a,b display the friction coefficients and forces of the two greases, which shows that St-C-MDI/ESO has lower friction forces and coefficients, indicating superior wear reduction, abrasion resistance, and lubricating performance compared to Li/MO. Figure 9c presents the optical wear scar images of the steel ball, which was lubricated with St-C-MDI/ESO and Li/MO; both samples have well-defined circular wear scars with clean edges. The wear scar diameter of St-C-MDI/ESO is 0.61 mm, which is smaller than that of Li/MO (0.64 mm), as shown in Figure 9d. The wear scar of Li/MO appears rougher, with more plowing marks, indicating less effective reduction in metal-to-metal contact during friction. This suggests that St-C-MDI/ESO forms a better adherent film on the metal balls, reducing wear [50,51].

### 3.4. Antioxidation Test

**Figure 10:**
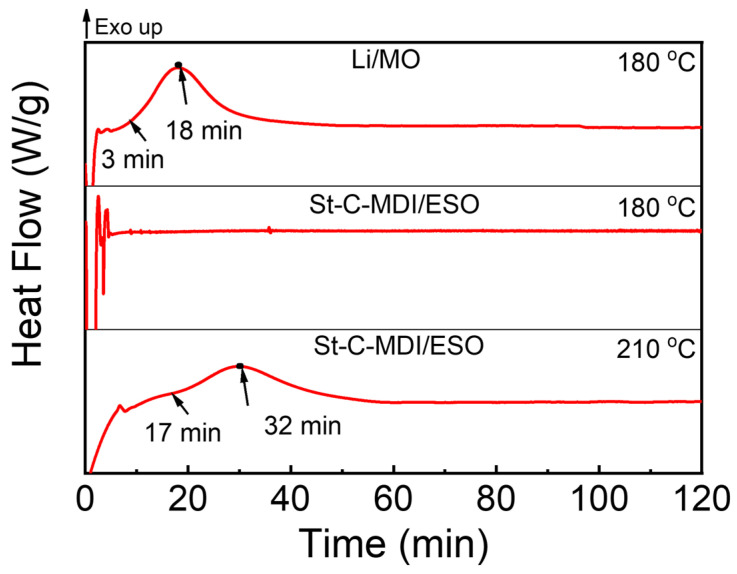
The oxidation induction time of Li/MO and St-C-MDI/ESO at 180 °C and St-C-MDI/ESO at 210 °C.

To investigate the antioxidant properties of different greases under high temperatures, the Oxidative Induction Time (OIT) was measured using differential scanning calorimetry (DSC) [16]. As shown in Figure 10, the Li/MO exhibited an OIT of 18 min at 180 °C, whereas no oxidation peak was observed for the St-C-MDI/ESO within the test period. When the temperature was increased to 210 °C, the St-C-MDI/ESO showed an OIT of 32 min. The results demonstrate that the straw-based grease possesses superior oxidation resistance. This is primarily attributed to the outstanding oxidation resistance of ESO [52].

### 3.5. Discussion

The outstanding tribological and rheological properties of the St-C-MDI/ESO grease originate from the effective chemical modification of cellulose. This modification promoted the formation of a stable three-dimensional network, as confirmed by SEM observations, which efficiently thickens the base oil and inhibits phase separation. The resulting grease displayed obvious shear-thinning behavior, which is consistent with a well-organized gel-like structure. Although its structural strength and recovery capability were lower than those of lithium grease (Li/MO), the formulated grease exhibited excellent lubricating performance, as evidenced by a considerably reduced friction coefficient (0.027) and smaller wear scar diameter (0.61 mm). The enhanced tribological behavior can be attributed to the improved adsorption of ESO onto the metal surface, promoting the formation of a more robust lubricating film that effectively minimizes friction and wear. Moreover, the exceptional oxidation induction time (OIT > 32 min at 210 °C) is mainly due to the inherent oxidative stability of the ESO base oil, in which epoxy functional groups suppress degradation pathways.

In contrast, Ilyin [53] developed a grease using microfibrillated cellulose (MFC) and triethyl citrate (TEC), reporting a friction coefficient ranging from 0.071 to 0.095. Borrero-López [54] formulated greases from wheat and barley straw in combination with castor oil, displaying friction coefficients between 0.091 and 0.095. Gallego [42] used acetylated chitosan as a thickener in castor oil and achieved friction coefficients in the range of 0.106 to 0.149. Similarly, Antonio M. Borrero-López [55] produced grease from cellulose pulp and castor oil, reporting a friction coefficient varying from 0.077 to 0.108. In another study, R. Sánchez [15] utilized chitin, chitosan, and their acylated derivatives as thickeners in soybean and castor oils, with friction coefficients observed between 0.12 and 0.25. Overall, the straw-based grease developed in this study demonstrates a comparative advantage in tribological performance. However, it should be noted that the current investigation is limited to a fundamental evaluation under standard test conditions. Future studies should focus on assessing its long-term stability, anti-corrosion properties, and performance under a wider range of temperatures and loads. Additionally, a life cycle assessment (LCA) of grease is recommended to quantitatively evaluate its environmental benefits.

## 4. Conclusions

This study extracted cellulose from corn straw and then modified it for use as a thickener to prepare a bio-based grease. Based on the tests performed on the bio-based grease, the following conclusions can be drawn:

1. The characterization of SEM, EDS, FTIR, and XRD verified that the MDI was successfully grafted onto the cellulose surface. TGA revealed that the thermal stability of the modified cellulose improved, with the char yield increasing from 12.5% to 14.8% at 800 °C compared to pure cellulose.
2. The bio-based grease, which was prepared by modified cellulose and ESO, exhibited a stable state that did not separate after being left to stand for 14 days.
3. The rheological tests demonstrated shear-thinning behavior of bio-based grease, which is consistent with the rheological characteristics of gel-like materials. The bio-based grease exhibits a weaker structure compared to the lithium-based grease.
4. The friction and wear tests revealed that the bio-based grease had lower friction coefficients and smaller wear scar diameters compared to commercial lithium-based greases, which indicates superior tribological properties.
5. The antioxidation test demonstrates that the St-C-MDI/ESO possesses superior oxidation resistance compared to the Li/MO, which is primarily attributed to the exceptional antioxidant performance of the ESO component.

## Figures and Tables

**Table 1:** TG data for St, St-C, St-C-MDI, ESO, and St-C-MDI/ESO.

Samples	T5%/°C	Tmax/°C	Yc/%
St	166.7	314.8	25.6
St-C	209.7	337.8	12.5
St-C-MDI	193.8	327.8	14.8
ESO	346.6	398.7	1.2
St-C-MDI/ESO	281.8	365.7	2.3

**Table 2:** Strain sweep and structural recovery data for St-C-MDI/ESO and Li/MO.

Samples	Flow Point/%	Destruction/%	Recovery/%
St-C-MDI/ESO	1.2	92.27	17.76
Li/MO	20	65.65	78.78

**Table 3:** Frictional properties of St-C-MDI/ESO and Li/MO.

Samples	Friction Coefficient	Friction Force/N	Wear Scar Diameter/mm
St-C-MDI/ESO	0.027 ±0.010	13.093	0.61 ±0.01
Li/MO	0.084 ±0.008	40.421	0.64 ±0.02

## Data Availability

The original contributions presented in this study are included in the article. Further inquiries can be directed to the corresponding authors.
